# Community-curated and standardised metadata of published ancient metagenomic samples with AncientMetagenomeDir

**DOI:** 10.1038/s41597-021-00816-y

**Published:** 2021-01-26

**Authors:** James A. Fellows Yates, Aida Andrades Valtueña, Åshild J. Vågene, Becky Cribdon, Irina M. Velsko, Maxime Borry, Miriam J. Bravo-Lopez, Antonio Fernandez-Guerra, Eleanor J. Green, Shreya L. Ramachandran, Peter D. Heintzman, Maria A. Spyrou, Alexander Hübner, Abigail S. Gancz, Jessica Hider, Aurora F. Allshouse, Valentina Zaro, Christina Warinner

**Affiliations:** 1 Department of Archaeogenetics, Max Planck Institute for the Science of Human History, Jena, 07745 Jena, Germany; 2 Institut für Vor- und Frühgeschichtliche Archäologie und Provinzialrömische Archäologie, Ludwig-Maximilians-Universität München, München, 80539 Germany; 3 Section for Evolutionary Genomics, GLOBE Institute, Faculty of Health and Medical Sciences, University of Copenhagen, Copenhagen, 1350 Denmark; 4 School of Life Sciences, University of Warwick, Coventry, CV4 7AL United Kingdom; 5 International Laboratory for Human Genome Research, National Autonomous University of Mexico, Queretaro, 76230 Mexico; 6 Section for GeoGenetics, GLOBE Institute, Faculty of Health and Medical Sciences, University of Copenhagen, Copenhagen, 1350 Denmark; 7 Microbial Genomics and Bioinformatics Research Group, Max Planck Institute for Marine Microbiology, Bremen, 28359 Germany; 8 BioArCh, Department of Archaeology, University of York, York, YO10 5DD United Kingdom; 9 Department of Earth Sciences, Natural History Museum, London, SW7 5BD United Kingdom; 10 Human Genetics, University of Chicago, Chicago, IL 60637 USA; 11 The Arctic University Museum of Norway, UiT The Arctic University of Norway, Tromsø, 9037 Norway; 12 Department of Evolutionary Genetics, Max Planck Institute for Evolutionary Anthropology, Leipzig, 04103 Germany; 13 Department of Anthropology, Pennsylvania State University, Pennsylvania, PA 16802 USA; 14 Department of Anthropology, McMaster University, Hamilton, L8S4L9 Canada; 15 McMaster Ancient DNA Centre, McMaster University, Hamilton, L8S4L10 Canada; 16 Department of Anthropology, Harvard University, Cambridge, MA 02138 USA; 17 Max Planck-Harvard Research Center for the Archaeoscience of the Ancient Mediterranean, Cambridge, MA 02138 USA; 18 Department of Biology, Università degli Studi di Firenze, Florence, 50122 Italy

**Keywords:** Metagenomics, Data publication and archiving, Genetic databases

## Abstract

Ancient DNA and RNA are valuable data sources for a wide range of disciplines. Within the field of ancient metagenomics, the number of published genetic datasets has risen dramatically in recent years, and tracking this data for reuse is particularly important for large-scale ecological and evolutionary studies of individual taxa and communities of both microbes and eukaryotes. AncientMetagenomeDir (archived at 10.5281/zenodo.3980833) is a collection of annotated metagenomic sample lists derived from published studies that provide basic, standardised metadata and accession numbers to allow rapid data retrieval from online repositories. These tables are community-curated and span multiple sub-disciplines to ensure adequate breadth and consensus in metadata definitions, as well as longevity of the database. Internal guidelines and automated checks facilitate compatibility with established sequence-read archives and term-ontologies, and ensure consistency and interoperability for future meta-analyses. This collection will also assist in standardising metadata reporting for future ancient metagenomic studies.

## Abstract (summary)

**Table:** 

Measurement(s)	genome • Metagenome • Metadata • Ancient DNA
Technology Type(s)	digital curation
Factor Type(s)	geographic location • sample age

Machine-accessible metadata file describing the reported data: 10.6084/m9.figshare.13241537

## Background & Summary

A crucial, but sometimes overlooked, component of scientific reproducibility is the efficient retrieval of sample metadata. While the field of ancient DNA (aDNA) has been celebrated for its commitment to making sequencing data available through public archives^1^, this data is not necessarily ‘findable’ (as defined in the FAIR principles^2^) - making the retrieval of relevant metadata time-consuming and complex. Metagenomic studies typically require large sample sizes, which are integrated with previously published datasets for comparative analyses. However, the current absence of standards in basic metadata reporting within ancient metagenomics can make data retrieval tedious and laborious, leading to analysis bottlenecks.

Ancient metagenomics can be broadly defined as the study of the *total* genetic content of samples that have degraded over time^3^. Areas of study that fall under ancient metagenomics include studies of host-associated microbial communities (e.g., ancient microbiomes^4^), genome reconstruction and analysis of specific microbial taxa (e.g., ancient pathogens^5^), and environmental reconstructions using sedimentary aDNA (sedaDNA)0^6^. Endogenous genetic material obtained from ancient samples has undergone a variety of degradation processes that can cause the original genetic signal to be overwhelmed by modern contamination. Therefore, to detect, quantify, and authenticate the remaining ‘true’ aDNA large DNA sequencing efforts are required^7,8^. These studies have only become feasible since the development of massively parallel ‘next-generation sequencing’, which enables the generation of large amounts of genetic data that are mostly uploaded to and stored on large generalised archives such as the European Bioinformatic Institute’s (EBI) European Nucleotide Archive (ENA, https://www.ebi.ac.uk/ena/) or the US National Center for Biotechnology Information (NCBI)’s Sequence Read Archive (SRA, https://www.ncbi.nlm.nih.gov/sra). However, as these are generalised databases used for many kinds of genetic studies, searching for and identifying ancient metagenomic samples can be difficult and time consuming, partly because of the absence of standardised metadata reporting for ancient metagenomic data. Consequently, researchers must resort to repeated extensive literature searches of heterogeneously reported and inconsistently formatted publications to locate ancient metagenomic datasets. Overcoming the difficulty of finding previously published samples is particularly pertinent to studies of aDNA, as palaeontological and archaeological samples are by their nature limited, and avoiding repeated or redundant sampling is of high priority^9–11^.

To address these issues, we established AncientMetagenomeDir, a CC-BY 4.0 licensed community-curated collection of annotated sample lists that aims to guide researchers to all published ancient metagenomics-related samples with publicly available sequence data. AncientMetagenomeDir was conceived by members of a recently established international and open community of researchers working in ancient metagenomics (Standards, Precautions and Advances in Ancient Metagenomics, or ‘SPAAM’ - https://spaam-community.github.io), whose aim is to foster research collaboration and define standards in analysis and reporting within the field. The collection aims to be comprehensive but lightweight, consisting of tab-separated value (TSV) tables for different major sub-disciplines of ancient metagenomics. These tables contain essential, sample-specific information for aDNA studies, including: geographic coordinates, temporal data, sub-discipline specific critical information, and public archive accession codes that guide researchers to associated sequence data (see Methods). This simple format, together with comprehensive guides and documentation, encourages continuous contributions from the community and facilitates usage of the resource by researchers coming from non-computational backgrounds, something common in interdisciplinary fields such as archaeo- and palaeogenetics.

**Fig. 1:**
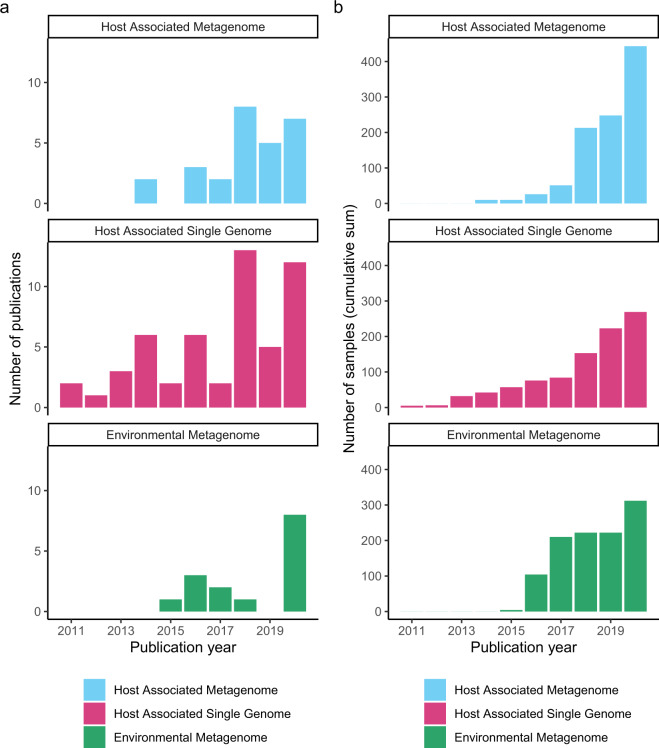
Timelines depicting the development of the sub-disciplines of ancient metagenomics as recorded in AncientMetagenomeDir as per release v20.09. (**a**) Number of ancient metagenomic publications per year. (**b**) Cumulative sum of published samples with genetic sequencing data or sequences in publicly accessible archives.

**Fig. 2:**
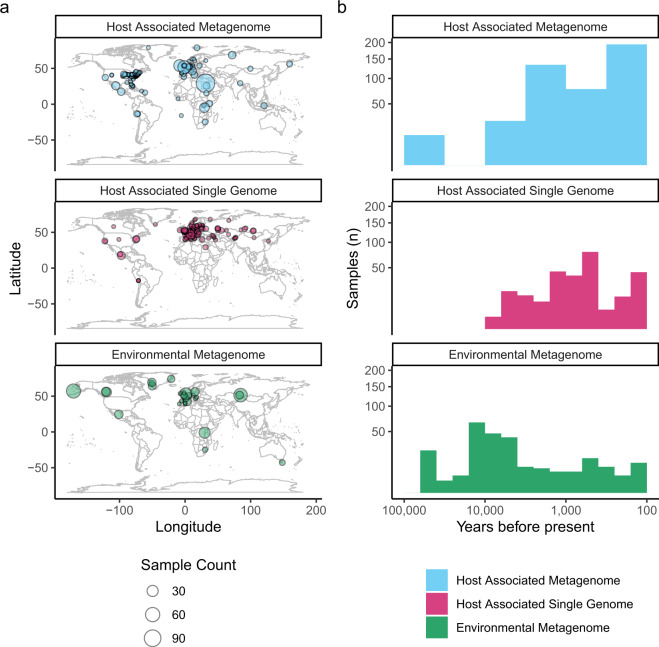
Summary of temporal and spatial information of ancient metagenomic samples as recorded in AncientMetagenomeDir v20.09. (**a**) Maps depicting the geographic distribution of samples for each sub-discipline. (**b**) Histogram of sample ages for each sub-discipline. For visualisation purposes, plot axes are log-scaled, bins calculated using the ‘Freedman–Diaconis’ rule, and only samples dated to younger than 50,000 years are displayed.

AncientMetagenomeDir is designed to track the development of ancient metagenomics through regular releases. As of release v20.09, this includes 87 studies published since 2011, representing 443 ancient host-associated metagenome samples, 269 ancient microbial genome level sequences, and 312 sediment samples (Fig. 1) spanning 49 countries (Fig. 2). We expect AncientMetagenomeDir to deliver three key benefits. First, it will contribute to the longevity of important cultural heritage by guiding future sampling strategies, thereby reducing the risk of repeated or over-sampling of the same samples or regions. Second, it can serve as a starting point for the development of software to allow rapid aggregation of actual data files and field-specific data processing. Third, it will assist in expanding meta-analyses (such as^12,13^) to a wider range of sample types and DNA sources in order to tackle broader palaeogenetic, ecological, and evolutionary questions. Finally, as a community-curated resource designed specifically for widespread participation, AncientMetagenomeDir will help the field to define common standards of metadata reporting (such as with MIxS checklists^14^), facilitating the creation of future databases that are consistent, and richer, in useful metadata.

## Methods

### Repository Structure

AncientMetagenomeDir^15^ is a community-curated set of tables maintained on GitHub containing metadata from published ancient metagenomic studies (https://github.com/SPAAM-community/AncientMetagenomeDir). While most submissions are made by SPAAM members, anyone with a GitHub account is welcome to propose (termed here ‘proposer’) and/or add publications for inclusion (termed ‘contributor’). Proposers and contributors can be (but do not have to be) authors of the original publication(s) proposed for inclusion. Submitted studies must be published in a peer-reviewed journal because the purpose of AncientMetagenomeDir is not to act as a quality filter and we do not currently make assessments based on data quality. The tables are formatted as tab-separated value (TSV) files in order to maximize accessibility for all researchers and to allow portability between different data analysis software.

Valid samples for inclusion currently fall under three sub-fields: (1) host-associated metagenomes (i.e., host-associated or skeletal material microbiomes), (2) host-associated single genomes (i.e., pathogen or commensal microbial genomes), and (3) environmental metagenomes (e.g., sedaDNA). In addition, a fourth category is currently planned: (4) anthropogenic metagenomes (e.g., dietary and microbial DNA within pottery crusts, or microbial DNA and handling debris on parchment). The definitions under which a sample is considered ‘ancient’ is adapted on a per sub-field basis. Generally, samples are required to have had reported evidence of hydrolytic damage at molecule termini, short fragment lengths, and contain fraction of non-endogenous content (e.g. as summarised in^3^). However, for example, due to regular use in ancient pathogenomics studies, samples preserved in long-term medical collections from the last century that have limited degradation may also be included. In the first release of AncientMetagenomeDir, we have specified a minimum age of older than 1950 CE. Samples must have been sequenced using a shotgun metagenomic approach, or alternatively a whole organelle- or chromosome-level enrichment approach, and sequence data must be publicly available on an established or stable archive. INDSC-associated repositories such as the EBI’s ENA or NCBI’s SRA and Genbank databases are preferred, as they are the most accepted and commonly used archives for raw sequencing data. However, DOI-issuing long-term archives (such as Zenodo or Figshare), institutional repositories (such as institutional data services), or field-specific established repositories (e.g., TreeBASE) can also be accepted. Data on personal or lab websites are not accepted due to uncertain storage longevity. We currently do not include laboratory negative controls, as we consider these to be ‘artefacts’ of lab procedures and better addressed with experiment-level metadata. If required by a researcher, controls can be identified via sample-associated project accession codes.

Publications included in the current release of AncientMetagenomeDir were selected for inclusion based on direct contributions by authors of publications and also from literature reviews of each sub-field made by the SPAAM community. In this process, a proposer initially suggests a publication to be included via a GitHub ‘Issue’. Publications may belong to multiple categories, and the corresponding issue is tagged with relevant category ‘labels’ to assist with faster evaluation and task distribution.

### Data acquisition

Members of the SPAAM community (termed ‘curators’) evaluate proposed publications for applicability under the criteria described above. Once approved, any member of the open SPAAM community can assign themselves to the corresponding Issue and will henceforth act as the contributor. A proposer from outside SPAAM who wishes to also be a contributor can be added to the SPAAM community by contacting a current member if desired. The contributor then creates a git branch from the main repository, manually extracts the relevant metadata from the given publication, and adds it to the assigned table (e.g., host associated metagenome, or environmental metagenome). Extensive documentation on submissions, including instructions on using GitHub, are available via tutorial documents and the associated repository wiki. Both are accessible via the main repository README under the ‘Contributing’ section. Furthermore, detailed documentation is also available to assist contributors and ensure correct entry of metadata, with one README file per table that contains column definitions and guidelines on how to interpret and record metadata.

The metadata in each table covers four main categories: publication metadata (project name, year, and publication DOI), geographic metadata (site name, coordinates, and country), sample metadata (sample name, sample age, material type, and (meta)genome type) and sequencing archive information (archive, sample archive accession ID). Due to inconsistency in the ways metadata are reported in publications and archives, and to maintain concise records, we have specified (standardised) approximations for the reporting of sample ages, geographic locations, and archive accessions, following MIxS^14^ categories where possible. This approach allows researchers using the dataset to access sufficiently approximate information during search queries to identify samples of interest (e.g. all samples from Italy dating from between 4500-2500 Before Present (BP), i.e., from 1950), which they can subsequently manually check in the original publication to obtain the exact dating information (e.g., Late Bronze Age, 3725+/−15 BP). Due to inconsistency in dating and reporting methods, dates are reported (where relevant) as uncalibrated years BP, and rounded to the nearest 100 years, due to the range of calculation and reporting methods (radiocarbon dating vs. historical records, calibrated vs. uncalibrated radiocarbon dates, etc.). We hope that future extensions of AncientMetagenomeDir will include more exact dating information, such as raw dates and radiocarbon lab codes, to allow for consistent calibration of whole datasets for more precise dating information. Geographic coordinates are restricted to a maximum of three decimals, with fewer decimals indicating location uncertainty (e.g., if a publication only reports a region rather than a specific site). For sequence accession codes, we opted for using *sample* accession codes rather than direct sequencing data IDs. This is due to the myriad ways in which data are generated and uploaded to repositories (e.g., one sample accession per sample vs. one sample accession per library; or uploading raw sequencing reads vs. only consensus sequences). We found that in most cases sample accession codes are the most straightforward starting points for data retrieval. However, we did observe errors in some data accessions uploaded to public repositories, such as multiple sample codes assigned to different libraries of the same sample, and insufficient metadata to link accessions to specific samples reported in a study. Overall, we found that heterogeneity in sample (meta)data uploading was a common problem, which highlights the need for improvements in both training and community-agreed standards for data sharing and metadata reporting in public repositories (such as an ancient metagenomic MIxS extension). In addition to metadata recorded across all sample types, we have added table-specific metadata fields to individual categories as required (e.g., species for single genomes and community type for microbiomes). Such fields can be further extended or modified with the agreement of the community.

### Data validation

After all metadata has been added, a contributor makes a Pull Request (PR) into the master branch. Every PR undergoes an automated ‘continuous-integration’ validation check via the open-source companion tool AncientMetagenomeDirCheck^16^ (https://github.com/SPAAM-community/AncientMetagenomeDirCheck, License: GNU GPLv3). This tool automatically checks each submission for conformity against a specification schema of minimum required information and formatting consistency (see Technical Validation). Usage of controlled vocabularies, alongside stable linking (via DOIs), within the specifications ensures reliable querying of the dataset, and allows future expansion to include richer metadata by linking to other databases. Descriptions for the minimum required fields for an AncientMetagenomeDir table are provided in Table 1.

**Table 1:** Core fields that are required for all AncientMetagenomeDir sub-discipline tables, including field type and standardised formatting description.

Field	Description	Field Type	Field Format
project_name	Unique AncientMetagenomeDir key for study	String	FirstAuthorYYYY
publication_year	Publication year of study	Integer	YYYY
publication_doi	Publication DOI (or library permalink)	String	Regex
site_name	Specific locality name where sample taken from	String	Free text
latitude	Latitude in decimal coordinate (WGS84 projection)	Number	Max. 3 decimals
longitude	Longitude in decimal coordinate (WGS84 projection)	Number	Max. 3 decimals
geo_loc_name	Present-day country name (INSDC) that locality resides in	String	Restricted enum
sample_name	Name of sample as reported in publication or archive	String	Free text
sample_age	Approximate date (before 1950, rounded to last 100 years)	Integer	YYYY
sample_age_doi	DOI of source of date. Can be more recent publication.	String	Regex
collection_date	Date of sampling of material for genetic analysis	Integer	YYYY
archive	Name of established data repository	String	Restricted enum
archive_accession	Sample-level accession code in data repository	String	Free text

Field formats are defined in a JSON schema, against which each new study submission is cross-checked by automated continuous integration (CI) checks and community peer-review. Further sub-discipline specific fields are included in the corresponding table, as required by the community.

Once automated checks are cleared, a contributor then requests a minimum of one peer-review performed by another member of the SPAAM community (termed ‘reviewer’). This reviewer checks the entered data for consistency against the table’s README file and also for accuracy against the original publication. Once the automated and peer-review checks are both satisfied, the publication’s metadata are then added to the master branch and the corresponding Issue is closed. For each added publication, a CHANGELOG is maintained to track the papers included in each release and to record any corrections that may have been made (e.g., if new radiocarbon dates are published for previously entered samples). The CHANGELOG or Issues pages on GitHub can be consulted to check whether a given publication has already been added (or excluded) from a table. Proposals and submissions can be made at any time, and contributed data is available on the main GitHub repository immediately after integration into the master branch. However, citable versions of the database are only made on each new (non-modifiable) release (see section Data Records). New submissions or corrections received after a release are included in subsequent versions.

## Data Records

**Fig. 3:**
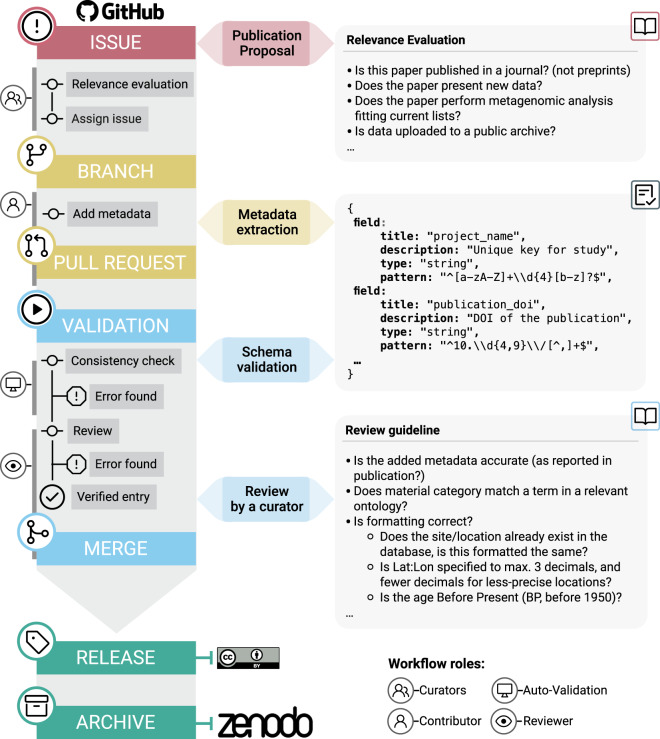
AncientMetagenomeDir submission and update workflow. The submission workflow is carried out on GitHub, and final releases are archived at Zenodo. Submissions go through both automated computational validation and also peer-review for consistency and accuracy.

AncientMetagenomeDir (https://github.com/SPAAM-community/AncientMetagenomeDir) and AncientMetagenomeDirCheck (https://github.com/SPAAM-community/AncientMetagenomeDirCheck) are both maintained on GitHub. AncientMetagenomeDir has regular quarterly releases, each of which has a release-specific DOI assigned via the Zenodo long-term data repository. Both the collection and tools are archived in the Zenodo repository with generalised DOIs^15^ and^16^, respectively. The full workflow can be seen in Fig. 3. Releases are made under a CC-BY 4.0 license (https://creativecommons.org/licenses/by/4.0/).

## Technical Validation

All data entries to AncientMetagenomeDir undergo automated continuous-integration validation prior to submission into the protected main branch. These tests must pass before being additionally peer-reviewed by other member(s) of the community (see section Data Validation). Automated continuous-integration (CI) validation tests consist of regex patterns to control formatting of specified fields (e.g. DOIs, project IDs, date formats), and cross-checking of entries against controlled vocabularies defined in centralised JSON schema, often derived from established term-ontologies. For example, valid country codes are guided by the International Nucleotide Sequence Database Collaboration (INSDC) controlled vocabulary (http://www.insdc.org/country.html), host and microbial species names are defined by the NCBI’s Taxonomy database (https://www.ncbi.nlm.nih.gov/taxonomy), and material types are defined by the ontologies listed on the EBI’s Ontology Look Up service (https://www.ebi.ac.uk/ols/index) - particularly the Uberon^17^ and Envo ontologies^18,19^. Entries must also have valid sample accession IDs corresponding to shotgun metagenomic, genome-enriched sequence data, or - when only available - consensus sequences, uploaded to established and stable public archives.

## Usage Notes

Usage of the resource typically consists of loading the TSV file of interest in software such as Microsoft Excel, LibreOffice Calc, or R. The data table can be subsequently sorted or queried to identify datasets of interest. It should be noted that certain metadata fields (e.g., sample_age, latitude, and longitude) are approximate and do not provide *exact* values; rather, if exact values for these fields are required, they must be retrieved from the original publication or requested from the publications’ authors. All selected data retrieved using AncientMetagenomeDir and used in subsequent studies should be cited using the original publication citation as well as AncientMetagenomeDir.

Retrieval of sequencing data using sample accession codes can be achieved manually via a given archive’s website, or via archive-supplied tools (e.g., Entrez Programming Utilities for NCBI’s SRA (https://www.ncbi.nlm.nih.gov/books/NBK179288/), or enaBrowserTools for EBI’s ENA (https://github.com/enasequence/enaBrowserTools).

Contributions to the tables are also facilitated by extensive step-by-step documentation on how to use GitHub and AncientMetagenomeDir, the locations of which are listed on the main README of the repository.

## Data Availability

An R notebook used for generating images with package versions can be found in the AncientMetagenomeDir repository at https://github.com/SPAAM-community/AncientMetagenomeDir/tree/master/assets/analysis (commit 4308bb7). Code for validation of the dataset (with version 1 used for the first release of AncientMetagenomeDir) can be found at https://github.com/SPAAM-community/AncientMetagenomeDirCheck and 10.5281/zenodo.4003826.
